# Physiological Changes and Interactions Between Microbiome and the Host During Pregnancy

**DOI:** 10.3389/fcimb.2022.824925

**Published:** 2022-02-21

**Authors:** Zain Zaki Zakaria, Shouq Al-Rumaihi, Rana S. Al-Absi, Huda Farah, Muram Elamin, Rahaf Nader, Salma Bouabidi, Sara Elgaili Suleiman, Shahd Nasr, Maha Al-Asmakh

**Affiliations:** ^1^ Department of Biomedical Sciences, College of Health Sciences, Qatar University (QU) Health, Qatar University, Doha, Qatar; ^2^ Biomedical Research Center, Qatar University (QU), Doha, Qatar; ^3^ Department of Biological and Environmental Sciences, College of Arts and Sciences, Qatar University (QU), Doha, Qatar

**Keywords:** microbiome, pregnancy, oral microbiota, gut microbiota, vaginal microbiota, probiotics, physiological changes

## Abstract

In recent years, it has become clear that microbiome play a variety of essential roles in human metabolism, immunity, and overall health and that the composition of these microbiome is influenced by our environment, diet, weight, hormones, and other factors. Indeed, numerous physiological and pathological conditions, including obesity and metabolic syndrome, are associated with changes in our microbiome, referred to as dysbiosis. As a result, it is not surprising that such changes occur during pregnancy, which includes substantial weight gain and significant changes in metabolism and immune defenses. The present review relates physiological changes during pregnancy to alterations in the microbial composition at various sites, including the gut, oral cavity, and vagina. Pregnancy has been linked to such microbial changes, and we believe that, in contrast to certain disease states, these microbial changes are vital for a healthy pregnancy, probably through their influence on the mother’s immunological, endocrinological, and metabolic status.

## Introduction

To ensure a healthy environment for fetal development, pregnancy is associated with pronounced changes in metabolism, hormonal status and immunological defenses, all of which may be influenced by microbiota resident in the gut, vagina, placenta and oral cavity (Nuriel-Ohayon et al., 2016). For example, changes in the endocrine system in response to maternal factors such as diet and usage of antibiotics influence the compositions of the gut and oral microbiome (Nuriel-Ohayon et al., 2019, Amir et al., 2020). Moreover, progesterone can increase the abundance of bifidobacterium in the gut (Nuriel-Ohayon et al., 2019; Amir et al., 2020).

Dysbiosis of the gut microbiome during pregnancy is associated with gestational diabetes, preeclampsia and restricted fetal growth. In addition, certain oral bacteria are pathogenic and can exert deleterious effects on the health and maturation of the fetus. Periodontal infections that are transmitted to other locations can lead to premature birth, low birth weight, and preeclampsia (Figuero et al., 2020) so that virtually all pregnant women require periodontal care (Rapone et al., 2020).

Here, we summarize recent research findings regarding alterations in microbiome during pregnancy.

## Physiological Changes During Pregnancy

To support the healthy growth and development of the fetus, pregnant women undergo changes in their endocrine, cardiovascular, respiratory, renal, and digestive systems.

### Changes in the Endocrine System

Already upon conception, the levels of certain hormones in a woman increase. Upon successful implantation of a fertilized egg into the uterine wall, placental trophoblasts begin to produce human chronic gonadotrophin (hCG) (Miller et al., 2020), the level of which rises during the first few weeks of pregnancy until it reaches its peak level of approximately 20000 mIU/mL during weeks 10-12, and thereafter, and at the end of the first trimester, falls steadily (Hendriks et al., 2019). hCG stimulates cells in the corpus luteum to start producing progesterone and estrogen, the levels of which increase as pregnancy progresses and the placenta grows, reaching their peaks during the third trimester (Sykes and Bennett, 2018). In addition, the hCG hormone is involved in the formation of vessels and the placenta, the differentiation of fetal cells and growth of fetal organs and preventing premature contractions of the uterus musculature (Barjaktarovic et al., 2017).

The many processes mediated by progesterone include the adaptation of the cervix for implantation of the fetus and differentiation of stromal cells into decidual cells. Furthermore, increasing progesterone levels prevent uterine contractions both by diminishing the levels of receptors for prostaglandin and oxytocin and directly inhibiting the contraction of resident smooth muscle cells (Sykes and Bennett, 2018). Rising estrogen levels are responsible for neoangiogenesis and the formation of tissues that become the placenta and support lactation (Noyola-Martínez et al., 2019). These hormonal changes cause the typical fatigue, nausea, constipation, and headaches associated with early pregnancy (Fuhler, 2020). Moreover, the growing placenta also produces hormones, including human placental lactogen (HPL), relaxin and human chronic gonadotrophin.

In addition, the thyroid gland of pregnant women may become more active, leading to hyperthyroidism (Kobaly and Mandel, 2019). Placental hormones such as lactogen promote insulin resistance, which is often accompanied by elevated production of insulin. Therefore, to avoid excessive plasma concentrations of glucose, pregnant women are often advised to pay attention to regulating their intake of carbohydrates (Mockridge and Maclennan, 2019)

### Circulatory and Cardiovascular Changes

A major cause of the cardiovascular changes associated with pregnancy is relaxation of the vascular smooth muscle in response to the increased circulating levels of estrogens, progesterone, and prostaglandins (Morton, 2021). Moreover, these hormonal changes lower the resistance of the pulmonary and systemic vessels and, thereby, blood pressure, which in most cases eventually returns to the non-pregnant value by the third trimester. Furthermore, the diastolic and stroke volumes and heart rate are elevated during pregnancy (Chen and Basso, 2007), resulting in a continuous increase in cardiac output, especially during the second and third trimesters (Morton, 2020). This increase, which can be as much as 50% during the third trimester, is targeted primarily to the placenta and uterus to nourish the growing fetus and the breasts in preparation for breastfeeding the newborn infant (Elkus and Popovich, 1992).

Furthermore, during the early phase of pregnancy, activation of the renin-angiotensin-aldosterone system causes sodium and water retention. Ultimately, this could expand the plasma volume and dilute the number of red blood cells (Claros et al., 2020), leading to anemia. In this context, the requirement for iron increases two- to three-fold, and many women experience iron deficiency during the second trimester of their pregnancy (Scholl and Reilly, 2000). The fetus requires this additional iron to synthesize hemoglobin and certain other enzymatic functions. In addition, capillary wedge and oncotic pressure reductions make pregnant women susceptible to pulmonary edema (Jarvis and Nelson-Piercy, 2020).

### Respiratory Changes

To fulfill the requirement of the developing fetus for oxygen, the respiratory system undergoes several physiological and anatomical changes, including elevated tidal volume, ventilation, and respiratory rate (Lemos et al., 2010; Hegewald and Crapo, 2011). At the same time, the expiratory reserve volume, total pulmonary capacity, and functional residual capacity all decline during gestation (Lemos et al., 2010), mainly due to alterations in the flexibility of ligaments in response to the higher levels of progesterone. In addition, the rise in intra-abdominal pressure as the uterus grows contributes considerably to these respiratory effects (Lee et al., 2017).

Moreover, this increase in ventilation causes the partial pressures of oxygen (paO_2_) and carbon dioxide (paO_2_) to be augmented and attenuated, respectively. These changes should, in turn, facilitate gas exchange across the placenta.

### Changes in the Renal System

During pregnancy, the renal system undergoes several physiological and anatomical adaptations designed to support the development of the fetus. One major change of this sort is enlargement and increased weight of the kidneys due to a larger interstitial volume and more extensive vasculature (Cheung and Lafayette, 2013). Moreover, due to the tremendous pressure exerted by the growing fetus (Cheung and Lafayette, 2013), the capacity of the mother’s bladder decreases, causing more frequent urination, one of the most common symptoms associated with pregnancy. In addition, renal blood flow may be elevated, increasing the glomerular filtration rate by as much as 50% and thereby reducing serum levels of creatinine (Park et al., 2017). Finally, both relaxation of smooth muscles in response to elevated progesterone levels and mechanical compression by the enlarging uterus can lead to retention of urine and hydronephrosis (Suarez et al., 2019).

As a result of the changes in the renal system during pregnancy this can also lead to deficiencies and raised levels of certain solutes. One change is hyponatremia which is caused by the high levels of hCG in pregnancy (Abbassi-Ghanavati et al., 2009). Another change is proteinuria due to protein excretion in urine being higher than normal. This is believed to be a result of multiple factors such as the increased Glomerular filtration rate (GFR), increased protein transport across the glomerular barrier as well as the decreased reabsorption of filtered protein (Kattah et al., 2017). Glucosuria is also another possible outcome, seen in around 50% of pregnant patients. It is mainly caused by decreased absorption of glucose in the proximal tubule (Alto, 2005).

### Changes in the Gastrointestinal System

Nausea, vomiting, heartburn, and constipation, the most common symptoms associated with pregnancy, are caused by changes in the gastrointestinal tract as the uterus enlarges (Herrero et al., 2001; Body and Christie, 2016). In addition, rising progesterone levels relax the lower esophageal sphincter, promoting reflux into the gastro-esophagus and, ultimately, heartburn (Jarvis and Nelson-Piercy, 2020). At the same time, pregnancy leads to constipation by slowing down gastric emptying. Furthermore, pregnant women are more prone to develop gallstones due to their elevated progesterone levels, which relaxes muscles, inhibits the release of cholecystokinin, and may thereby result in biliary stasis (Mockridge and Maclennan, 2019).

### Changes in the Hematologic System

**Figure 1:**
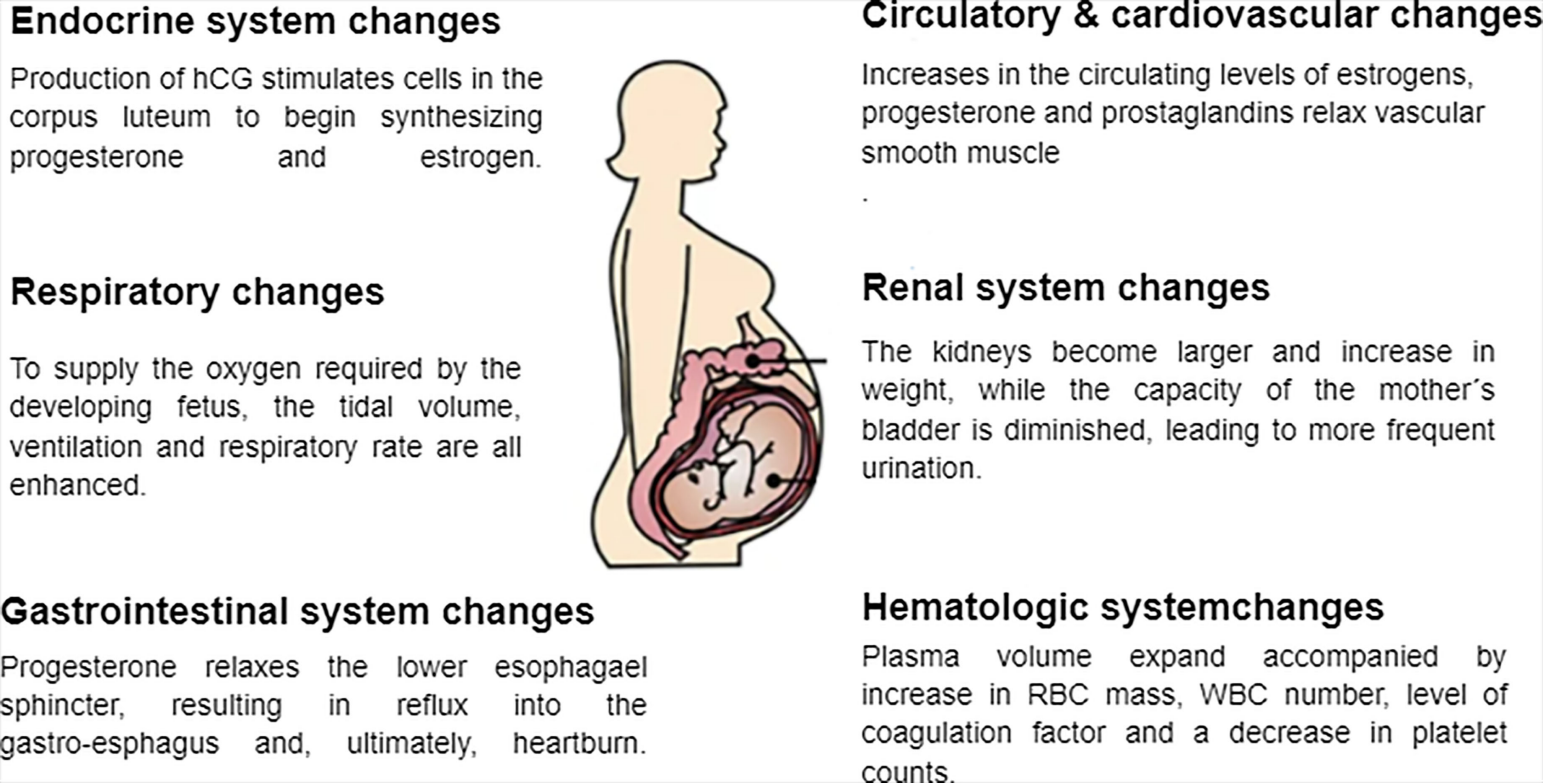
Physiological changes associated with pregnancy.

As with every other system in the body, the hematologic system also experiences changes during pregnancy. The most significant changes include expanded plasma volume accompanied with a higher red blood cell mass that leads to physiologic anemia as mentioned earlier (Bernstein et al., 2001). Additionally, during the first trimester white blood cells increase due to the physiological stress that pregnancy places on the body. This is especially evident with neutrophils. The most likely reason for this is the lack of neutrophilic apoptosis ability during pregnancy as a result of an increase in inhibitory factors in the serum. In addition, there is an increase in the number of myelocytes and metamyelocytes which indicate the increased level of activity in the bone marrow and erythropoiesis during pregnancy. In the first and second trimesters there is also a decrease in the lymphocyte count, compared to an increase in the third trimester (Chandra et al., 2012; Al-Shafei et al., 2020). Studies have also shown that platelet counts decrease in pregnancy, mainly in the third trimester. This is known as gestational thrombocytopenia, which occurs as a result of the hemodilution present in pregnancy accompanied with increased platelet activation and clearance. Pregnancy is also associated with an increase in the levels of coagulation factors due to the increase in estrogen levels during this time, resulting in a prothrombotic state, especially in the third trimester (Patel and Balanchivadze, 2021). **Table 1** and **Figure 1** summarize the physiological changes associated with pregnancy.

**Table 1:** Physiological changes during pregnancy.

**Cardiovascular system**	Decrease peripheral vascular resistance Increased heart rate Decreased arterial pressure Increased cardiac output Increase in total body water, capillary hydrostatic pressure, and blood volume	(Sanghavi and Rutherford, 2014; Furfaro et al., 2018)
**Respiratory system**	Mucosal changes in the upper airway include edema, hyperemia, leakage of plasma into the stroma, glandular hypersecretion, increased mucopolysaccharide content and increased phagocytic activity Increased tidal volume Decreased residual volume Increased minute-ventilation by 30-40% increased respiratory center simulation → increased respiratory rate Decreased PaCO2	(Hegewald and Crapo, 2011; Cordioli et al., 2013)
**Gastrointestinal system**	Decreased muscle tone across the digestive tract Delayed gastric emptying and diaphragm elevation by the pregnant womb Increase in gastric PH and reduced gastrointestinal motility Increased production of pro-inflammatory cytokines by Kupffer cells Changes in bile composition	(Cordioli et al., 2013; Furfaro et al., 2018; Gomes et al., 2018)
**Renal system**	The glomerular filtration rate increases by 50% Decrease in serum urea, creatinine, and uric acid values Ureteropelvic dilation and decreased ureteral pressure due to smooth muscle relaxation Increased intravesical pressure due to the pregnant uterus weight Increased renal plasma flow and vesicoureteral reflux Asymptomatic bacteriuria Flaccid bladder	(Cordioli et al., 2013; Cheung and Lafayette, 2013)
**Genital system**	Decreased vaginal Ph Increased glycogen in vaginal epithelium Increased uterine blood flow and the vascular bed proliferates Uterus increases in size to contain the growing fetus	(Parry and Vodstrcil, 2007; Cordioli et al., 2013)
**Hematologic system**	Increases factors VII, VIII, IX, X, XII, Von Willebrand and fibrinogen Decreased fibrinolytic activity Decreased protein S Increased plasma and red cell volume Anemia	(Cordioli et al., 2013)

## Changes in Microbiome Associated With Pregnancy

### The Gut Microbiome

The gastrointestinal tract is colonized by various microorganisms, including bacteria, protozoa, viruses and archaean and the composition of this microbiome varies with age. In adult humans, approximately 80% of this microbiome consists of bacteria belonging to the phyla Actinobacteria, Firmicutes and Bacteroidetes (Baldassarre et al., 2018). In the case of the neonate, colonization of the gut, which occurs during and after birth, is affected by mode of delivery and breastfeeding. The neonatal gut is colonized immediately for the most part by *Enterococci*, *Staphylococci*, and *Enterobacteria*; during the first days of post-natal life, *Lactobacillus*, *Clostridium*, *Bifidobacterium*, and *Bacteroides* take up residence; and the gut composition of bacteria become similar to that of adults at one year of age (Baldassarre et al., 2018).

Most pregnancies progress without incident. But approximately 8 percent of all pregnancies involves deleterious fetal and maternal health complications, the most common being preterm birth, diminished intrauterine growth, preeclampsia, and eclampsia (Maresh, 1990). The gut microbiota associated with normal and complicated pregnancies differ and the physiological changes, including the rise in the progesterone level, affect the composition of the maternal microbiome. More specifically, the microbiome in pregnant women contains a larger proportion of, in particular, *Bifidobacterium*, but also of *Proteobacteria* and *Actinobacteria* (Koren et al., 2012). During the third trimester alpha diversity is reduced, while both beta diversity and the abundance of opportunistic pathogens are elevated.

Some researchers have concluded that these changes are temporary and of no significance (DiGiulio et al., 2015; Yang et al., 2020), whereas others have found that insertion of human gut microbes from the third trimester into mice leads to weight gain and more pronounced low-grade inflammation (Turjeman et al., 2021). Furthermore, all alterations in the gut microbiome directly influence maternal metabolism (Paul et al., 2018), which, in turn, impacts the development and growth of fetal organs (Jiang et al., 2021).

In addition, the composition of the gut microbiome may be altered by poor dental health or inflammatory bowel disease in a manner linked to an increased risk for spontaneous premature delivery. In one investigation, the beta diversity of the maternal gut microbiota was found to differ between those who delivered preterm and normal deliveries (Hiltunen et al., 2021). In another case, mothers who underwent spontaneous preterm delivery exhibited lower diversity in their gut microbiome, particularly with respect to *Bifidobacterium* and *Streptococcus* (Dahl et al., 2017).

Moreover, gut microbes can give rise to intrauterine infection, as reflected in the presence of these microbes in the amniotic fluid of women who experienced premature rupture of membranes (Edwards et al., 2017). The mechanism by which gut microbes move to the uterus is not yet known, but two possible mechanisms have been proposed: the first is that gram-negative bacteria, which express lipopolysaccharide that elicits the production of prostaglandins and other mediators of inflammation, ascend *via* the vagina; and the second, that the content of the gut leaks into the uterus or placenta (Edwards et al., 2017).

### The Oral Microbiome

The healthy human oral cavity contains approximately 50-100 million bacteria belonging to 700 species (Kilian et al., 2016), including *Lactobacilli, Staphylococci, Streptococcus* (Dewhirst et al., 2010; Kumar et al., 2013; Saadaoui et al., 2021). The composition of this complex community is affected by several factors, such as nutrition, oxygen levels, and pH (Saadaoui et al., 2021). Imbalances in the oral microbiome have been found to be associated with certain diseases, as well as with pregnancy. Imbalances in the oral microbiota, particularly during pregnancy, have been linked to a variety of disorders (Farrell et al., 2012). In fact, the oral microbiome of a healthy pregnant woman and a pregnant woman with certain diseases, e.g., gestational diabetes, differ (Li et al., 2021).

The changes in microbiome that occur in connection with pregnancy include the microbiome in the oral cavity (Saadaoui et al., 2021; Turjeman et al., 2021). For instance, the microbiome detected in saliva differs between pregnant and non-pregnant women, with the former showing an abundance of, e.g., *Porphyromonas*, *Treponema* and *Neisseria*, while in the latter, *Veillonella* and *Streptococcus* were overrepresented (Lin et al., 2018). The oral microbiome of pregnant women contain high numbers of bacteria, mainly during the first trimester (Fujiwara et al., 2017) including *Porphyromonas, Neisseria*, and *Treponema* (Lin et al., 2018) and certain pathogenic bacteria (Chong et al., 2018). Moreover, certain specific species of bacteria, such as *Staphylococci, Streptococci, Aggregatibacter actinomycetemcomitans*, and *Porphyromonas gingivalis*, are more abundant in the oral microbiome during the first and second trimesters of pregnancy. During pregnancy, the proliferation and growth of *Streptococcus, Lactobacillus, Escherichia coli*, and *Bifidobacterium* species vary (Pelzer et al., 2012).

Furthermore, the hormonal changes that pregnant women undergo promote the formation of bacterial plaque, thereby resulting in gingivitis, especially during the second to third trimesters (de Souza Massoni et al., 2019), which causes complications of pregnancy such as preeclampsia, preterm birth (PTB), low birth weight, and miscarriage (Cobb et al., 2017). The amniotic fluid of a woman who went into preterm labor contained *Fusobacterium nucleatum*, suggesting that oral bacteria can translocate to the placenta (Nuriel-Ohayon et al., 2019; Amir et al., 2020). In another woman who suffered from gingivitis and gave an unusual full-term stillbirth, *Fusobacterium nucleatum* was detected in both the placenta and newborn infant, indicating that this bacterium originated from the maternal subgingival plaque (Nuriel-Ohayon et al., 2019; Amir et al., 2020). It appears possible that the environments in the oral cavity and placenta contain similar factors that promote colonization and growth of *Fusobacterium nucleatum* (Nuriel-Ohayon et al., 2019; Amir et al., 2020).

In addition, a positive correlation between the presence of a periodontopathogen (*Porphyromonas gingivalis*) and progesterone levels in the first trimester of pregnancy was observed (de Souza Massoni et al., 2019). Other studies confirmed the growth of certain gram-negative anaerobic bacteria, including *Prevotella nigrescens*, *Campylobacter rectus* (Yokoyama et al., 2008; Gürsoy et al., 2009), and *Prevotella intermedia* (Muramatsu and Takaesu, 1994), which is promoted by the hormonal changes that occur during pregnancy. Moreover, high estrogen levels promote infection by *Candida* (Kumar et al., 2013; Fujiwara et al., 2017).

Willmott and co-workers (2020) demonstrated that the composition of the oral microbiome accurately reflects the dietary content of nitrate and the healthy regulation of blood pressure (Willmott et al., 2020). Bacteria in the oral cavity, located primarily on the tongue’s surface, reduce nitrate enzymatically, resulting in the presence of nitrite in the saliva, which is subsequently transformed in the stomach into nitric acid and then reduced to nitric oxide (NO). This process is related to blood pressure in two different ways: the plasma level of nitrate is related inversely to blood pressure, and, at the same time, NO is a key signal molecule in connection with processes that regulate the circulatory system (Willmott et al., 2020).

Nitric Oxide (NO) is one of the reaction products produced by nitric oxide synthase (NOS) enzymes that catalyze NADPH and tetrahydrobiopterin (BH4)-dependent oxidation of L-arginine to L-citrulline (Ghimire et al., 2017). Nitric oxide serves in a wide aspect of human physiology and it takes part in vasodilation, endothelial function, mitochondrial function, prevention of platelets aggregation, neurotransmission, immune defense, and metabolism (Hezel and Weitzberg, 2015). In research from Walker et al., (2018) it was indicated that The oral microbiome play an important role in the production of the nitric oxide (NO) through nitrate-nitrite-NO pathway. The oral microbiota is deficient in the NO enzymes responsible for yielding the catalyzed NO from L-arginine (Qu et al., 2016; Willmott et al., 2020). According to Walker et al., (2018), the production of nitric oxide occurs in the human body by two methods: dependent and independent. Furthermore, the microbiome in the human body produces NO in an independent way through fermentation depending on food intake by the consumption of nitrate (NO3^-^) and nitrite (NO2^-^). In addition, oral microbiota has a major role in cardiovascular system wellbeing and in the regulation of blood pressure, including in pregnancy (Willmott et al., 2020). A case-control study in a tertiary facility in Ghana (Darkwa et al., 2018) found that the level of serum nitric oxide rises rapidly during pregnancy and peaks during the third trimester of a healthy pregnancy. However, other research found that the progressive rise in serum nitric oxide levels during pregnancy is not significant. According to (Zullino et al., 2018), nitric oxide is the principal vasodilator in the placenta, making it crucial to several physiological functions of an uncomplicated pregnancy. Placental perfusion, platelet adhesion and aggregation in the intervillous space, and fetoplacental vascular response are all regulated by NO during implantation, early embryonic development, and feto-placental vascular reaction (Zullino et al., 2018).

### The Vaginal Microbiome

The composition of the vaginal microbiome, which plays an essential role in both maternal and fetal health (Nelson et al., 2016), can be altered by many different factors, including hormones, sexual practices, pregnancy, hygiene, urogenital infections and pharmacological treatments (Kroon et al., 2018; Noyes et al., 2018; Parolin et al., 2018). Typically, *Lactobacilli* predominate and, together with other bacterial species, maintain a pH of 3.8-4.5 (Mendling, 2016). The vaginal environment changes dramatically during pregnancy, resulting in an even greater abundance of *Lactobacillus* spp. and pronounced changes in the metabolic profiles of the bacteria present (Marangoni et al., 2021). Complicated pregnancies and preterm birth are associated with less *Lactobacilli* and a greater variety of bacteria (Di Simone et al., 2020).

Several investigations into a potential link between the composition of the vaginal microbiome and miscarriage have revealed that miscarriages during the first trimester appear to associate with lower levels of *Lactobacillus* spp. and more pronounced alpha diversity. The presence of pathogenic microorganisms raises the risk for infections such as bacterial vaginosis, which has been linked to premature rupture of membranes and preterm birth. A meta-analysis concluded that, even after controlling for other major risk factors, the risk of preterm delivery in women with bacterial vaginosis caused by, e.g., *Prevotella bivia*, *Peptostreptococcus*, and/or *G. vaginalis* increased more than two-fold (Leitich et al., 2003). Moreover, a higher risk for preterm birth rate was correlated with the presence of specific vaginal fungi such as *Candida albicans* (Neuman and Koren, 2017) and variations in the vaginal pH caused by changes in the microbiome (Mysorekar and Cao, 2014).

**Figure 2:**
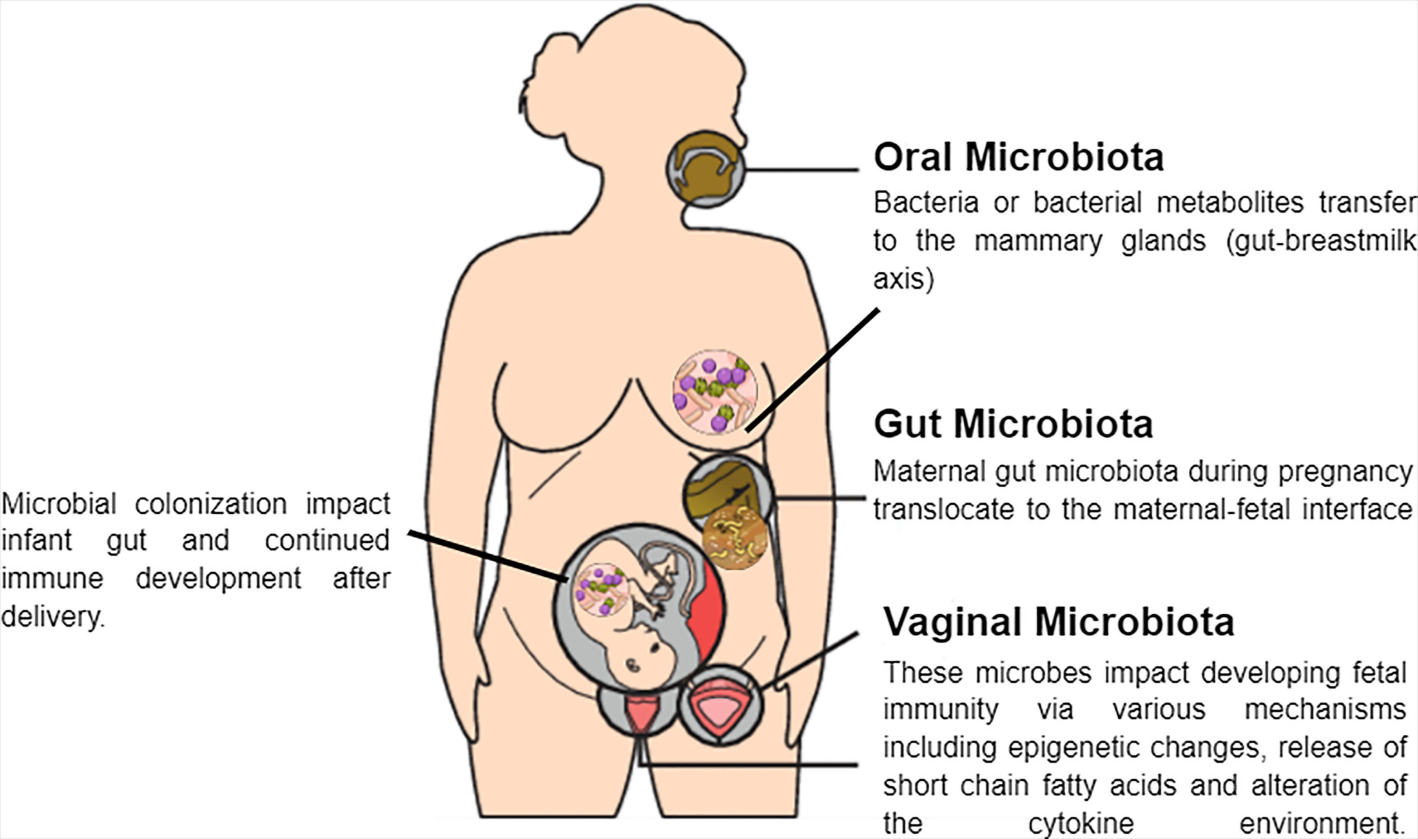
Potential mechanisms of crosstalk between maternal microbiota and the fetus.

In addition to the factors mentioned above, the composition of the vaginal microbiota is influenced by ethnic background and genetic polymorphisms that affect the ability to produce anti- or pro-microbial substances. Such polymorphisms are present in the genes that encode the antagonist of the interleukin 1 (IL-1) receptor and the Toll-like receptor (TLR) 4, which acts in the innate recognition of Gram-negative bacteria and can influence individual susceptibility to complications during pregnancy (Mendling, 2016). Furthermore, women who have embryonic miscarriages exhibit higher vaginal levels of interleukin 2 (IL-2) and lower levels of interleukin 10 (IL-10) than control subjects (Xu et al., 2020). **Table 2** summarize the change in the maternal microbiome during pregnancy. And **Figure 2** shows the potential mechanisms of crosstalk between maternal microbiota and offspring immunity.

**Table 2:** List of organisms and their associated effects during pregnancy.

Causative microbiome	Effects on pregnancy	Reference
**The gut microbiome**		
*Bifidobacteria*	Atopic eczema and asthma	(Baldassarre et al., 2018)
*Enterobacteriaceae*	Preterm newborns	(Baldassarre et al., 2018)
*Enterococcaceae*		
*Proteobacteria*	Metabolic syndrome such as weight gain, hyperglycemia, insulin resistance.	(Koren et al., 2012)
*Actinobacteria*		
*Prevotella*	Protection against food allergies and Gestational diabetes	(Koren et al., 2012; Turjeman et al., 2021)
*Staphylococcus spp*,	Weight gain	(Turjeman et al., 2021)
*Escherichia coli*		
*Ruminococcaceae*, and *Collinsella*	Gestational diabetes	(Turjeman et al., 2021)
*Enterobacteriacea*e, *DesulfovibrioParabacteroides distasonis*		
*Bulleidia moorei*,	Risk of Preeclampsia	(Turjeman et al., 2021)
*Clostridium perfringens*,		
*Fusobacterium and Veillonella*		
*Planococcaceae, Lactobacillaceae and Enterobacteriaceae*	Preterm neonates	(Hiltunen et al., 2021)
*Bifidobacterium*	Preterm neonates	(Dahl et al., 2018)
*Streptococcus*		
*Listeria monocytogenes*	Still birth	(Edwards et al., 2017)
**The oral microbiome**		
*Pg and intrauterine Bergeyella*	Delivered prematurely	(Saadaoui et al., 2021)
*Lautropia* and *Neisseria*	Saliva and dental plaque	(Li et al., 2021)
*Porphyromonas gingivalis*	Periodontal inflammation, placentas of patients with preeclampsia	(Cobb et al., 2017; Lin et al., 2018)
*Filifactor alocis*	Placentas of patients with preeclampsia	(Cobb et al., 2017)
*Fusobacterium nucleatum*	Preterm birth, periodontal disease and adverse pregnancy complications	(Cobb et al., 2017; Amir et al., 2020; Saadaoui et al., 2021)
*Prevotella nigrescens*	Pregnancy gingivitis	(Gürsoy et al., 2009)
*Genera Rothia and Staphylococcus*	Oral nitrate reduction	(Willmott et al., 2020)
*Prevotella*	Oral nitrate reduction	(Willmott et al., 2020)
**The vaginal microbiome**		
*Lactobacilli*	Pregnancy-related complications and preterm birth	(Di Simone et al., 2020)
*Prevotella bivia, Peptostreptococcus*, and/or *G. vaginalis*	Preterm delivery	(Leitich et al., 2003)
*Candida albicans*	Preterm delivery	(Neuman and Koren, 2017)

## The Effect of Probiotics on Microbiome During Pregnancy

The World Health Organization (WHO) defines probiotics as live microorganisms that, upon administration through the diet or as a supplement, have beneficial effects on the host (Buggio et al., 2019). A variety of studies indicate that by regulating the gut and vaginal microflora, the right combination of probiotics (Baldassarre et al., 2018) can lessen the risk of pregnancy complications such as preterm birth (Arango et al., 2015). It was proposed recently that probiotics prevent preterm birth by altering the composition and function of the gut microbiome to improve maternal glucose metabolism (Buggio et al., 2019).

However, findings in this area are controversial. For instance, one study that included 4098 women found that probiotics can either decrease or increase the risk for birth between weeks 34-37 of pregnancy (Buggio et al., 2019), supporting the proposal that more detailed investigations on the types of probiotic, the characteristics of individual women and length of administration are required (Jarde et al., 2018). In another case, probiotics provided no protection and were shown to be capable of initiating the inflammatory cascade associated with preterm birth (Othman et al., 2007; Arango et al., 2015). In contrast, dietary supplementation with probiotics has been reported to reduce the abundance of *Bifidobacterium* and attenuate the increase in *Atopobium vaginae* that is linked with more than 70% of cases of bacterial vaginosis, which causes preterm birth (Baldassarre et al., 2019). At the same time, it appears that following antibiotic treatment, supplementary probiotics can lower the vaginal pH to an optimal value hence promoting the restoration of vaginal microbiota, thereby preventing the reduction in levels of anti-inflammatory cytokines (Baldassarre et al., 2019).

Moreover, the administration of probiotics during pregnancy has been shown to raise the levels of anti-inflammatory molecules such as IL-10 and TGF-B in breast milk, which aids in the maturation of the infant’s gut by stimulating the secretion of IgA and oral tolerance (Baldassarre et al., 2018). Furthermore, probiotics can effectively prevent allergic reactions. The lymphoid system of the newborn is not fully developed, with a limited Th1 response, and the microbiota plays an essential role in bridging this gap between Th1 and Th2 responses. Accordingly, alteration of the gut microbiota that results in loss of this modulation of inflammatory cytokines can augment the risk for development of atopic eczema (Baldassarre et al., 2018).

More than 15% of young infants suffer from gestational disorders such as infantile colic. Probiotics can influence the pathogenesis of such disorders by altering the composition of the gut microbiome, which is involved in direct bidirectional relationships with the brain. The three mechanisms proposed as explanations for this influence by probiotics include alterations in the secretion of cytokines and chemokines, involvement of microbiota in neural pathways, and stimulation of the intestinal neuroendocrine pathway. Interestingly, the usage of a mixture of probiotics has been found to reduce crying by infants with colic during breastfeeding (Baldassarre et al., 2018), and probiotics appear to significantly impact the composition of the neonatal microbiota. It has been suggested that probiotics such as *Lactobacillus* can interact directly with the host *via* pattern recognition receptors (PRR), for which the peptidoglycan in the wall of gram-positive bacteria and lipopolysaccharide of gram-negative bacteria serve as ligands (Devi et al., 2021). The direct interaction involves the binding of probiotic bacteria to these receptors on the host’s dendritic and intestinal epithelial cells, thereby preventing cytokine-induced apoptosis and production of defensins and mucus (Devi et al., 2021).

Another proposed mechanism involves the generation of toxic or antimicrobial compounds such as bacteriocin by probiotics (Oktaviyani et al., 2021). For example, *Lactobacillus crispatus* F177 and *Lactobacillus paracasei* F2 and F28 produce hydrogen peroxide, which suppresses the growth of *Staphylococcus aureus*. In addition, probiotics compete with pathogenic species for adhesion to the surface of intestinal epithelial cells. Adhesion molecules such as a mucus-binding protein on the surface of probiotics facilitate their interaction with host dendritic cells, thus enhancing the phagocytic capacity of these cells (Devi et al., 2021).

## Conclusions

In this review, we describe the changes in the compositions of the gut, oral and vaginal microbiome that occur in connection with pregnancy. The hormonal, immunological and metabolic changes that pregnant women undergo influence these compositions and vice-versa, appropriate adaptation is required to support optimal fetal growth and development.

Imbalances in the microbiota can lead to complications of pregnancy such gestational diabetes, preterm delivery and preeclampsia. Manipulating microbiome composition during pregnancy through probiotics could result in improved maternal health and pregnancy outcomes. Maternal micorbioem and fetal interaction during pregancy is critical for fetal developemt. 

The mechanisms by which these microbiome and the host interact during pregnancy and regulation of these interactions require elucidation.

## Author Contributions

Conceptualization, ZZ & MA-A; writing—original draft preparation, SA-R, RA, HF, ME, RN, SB, SS, and SN; Figure design and tables, ZZ; writing—review and editing ZZ & MA-A; funding acquisition, MA-A. All authors contributed to the article and approved the submitted version.

## Funding

This research was funded by Qatar National Research Fund (QNRF), grant number UREP26-104-3-044.

## Conflict of Interest

The authors declare that the research was conducted in the absence of any commercial or financial relationships that could be construed as a potential conflict of interest.

## Publisher’s Note

All claims expressed in this article are solely those of the authors and do not necessarily represent those of their affiliated organizations, or those of the publisher, the editors and the reviewers. Any product that may be evaluated in this article, or claim that may be made by its manufacturer, is not guaranteed or endorsed by the publisher.
